# Supplementary material for: Maternal biomarkers of endothelial dysfunction and pregnancy outcomes in women with and without HIV in Botswana
Source: PLoS One. 2023 Feb 23;18(2):e0281910. doi: 10.1371/journal.pone.0281910 (PMC9949641; doi:10.1371/journal.pone.0281910)
Supplement: Attachment — Submitted filename: PONE-D-21-20063 - Response to Reviewers.docx [file pone.0281910.s002.docx]

Editor in Chief

PLOS one Journal

Dear Emily Chenette,

**RE: PONE-D21-20063 “Maternal biomarkers of endothelial dysfunction and pregnancy outcomes in women with and without HIV in Botswana”**

Thank you for the response given to our manuscript titled “*Maternal biomarkers of endothelial dysfunction and pregnancy outcomes in women with and without HIV in Botswana”.*  We have revised the manuscript by addressing point-by-point the comments made by the sole reviewer.

Gaerolwe Masheto

Corresponding Author

Reviewer's Responses to Questions

**Reviewer Comments #1.1**: The authors have taken advantage of stored plasma samples and data collected from an observational study (Tshipidi) that enrolled in HIV-positive and HIV-negative pregnant women in Botswana 2010-2012 (a time when ART regimen in pregnancy was determined by CD4 count), following the participants for 2 years after enrollment, to try to assess whether elevation in three biomarkers of endothelial dysfunction may be in the causal pathway for increased risk of adverse pregnancy outcomes in HIV-infected women. This short report found only one marker of endothelial activation, VCAM-1, was elevated in HIV infection and with no/recent ART, and none of the markers were associated with birth outcome (stillbirth, SGA). Unfortunately, there were only limited samples remaining on the comparative HIV-negative women (9% vs 82% for HIV positive women), which could potentially restrict the power of the study to determine differences by HIV status.

**Authors Response 1.1:** We agree with the reviewer and acknowledge this limitation in the discussion. We believe these data can help inform further studies of potential mechanisms that could explain differences in pregnancy outcomes between women living with HIV and those not living with HIV (and by ART regimen).

**Reviewer Comments #1.2**: Page 13, line 156: Table 1: Consider adding the “N” in the first row under the column titles so it is clear to reader.

**Authors Response 1.2:** Table 1 has been updated to include the “N” as suggested.

**Reviewer Comments #1.3**: Page 15, top paragraph lines 162-165, 170-172: Consider putting in the actual median values for the various markers (log ng/mL) as opposed to just listing the p values. Figure 1 on page 23 does not provide values, leaving the reader to guess what the median is in the comparative groups. A comment that while statistically significantly different by HIV status for VCAM-1 and ICAM-1, visually the values do not look extremely different. For example, in figure 1 for VCAM-1, the median value looks like ~2.8 in HIV+ vs 2.6 log10 ng/mL in HIV- women. The clinical significance of such differences is not clear to me.

**Authors Response 1.3:**

Figure 1 and Figure 2 have been updated include actual mean values for the various markers (log ng/mL) and text updated as below**.**

The text in lines 162 to 165 has been updated to as follows: “Fig 1 shows the levels of VCAM-1, ICAM-1 and e-selectin by maternal HIV status. WLHIV had a statistically significantly higher mean VCAM1 (Mean 2.90; 95% Confidence Interval(CI):2.88-2.93) than women without HIV (Mean 2.77; 95%CI: 2.72-2.83), Fig 1a. Women without HIV had a statistically significantly higher mean ICAM1 (Mean 2.42; 95%CI:2.40-2.44) compared to WLHIV (Mean 2.50;95% CI: 2.44-2.55), Fig 1b.”

The text in lines 170 – 172 has been updated to as follows: “ICAM-1 and e-selectin levels were not significantly different by the timing of ART relative to sample collection (Fig 2b, c). However, women starting ART during pregnancy had statistically significantly higher mean log10 VCAM1 levels (Mean 2.92; 95%CI:2.88-2.95) than women who were on ART before conception, in both the samples that were collected before (Mean 2.83; 95%CI: 2.77-2.88;p=0.02) or after (Mean 2.91; 95%CI: 2.87-2.944;p=0.03) ART initiation, Fig 2a.

**Reviewer Comments #1.4.** Discussion:

As noted above, the clinical significance of the differences observed is not clear. The authors note that “elevated VCAM-1” in the general population is associated with endothelial dysfunction. However, what levels define “elevated” that are associated with endothelial dysfunction is not discussed. Whether a decimal point difference in levels of the marker between groups (from visual inspection of the graphs) means that the marker is elevated enough to be associated with endothelial dysfunction is not clear. Additionally, references 23, 24 (referred to in line 202) report that while ICAM-1 was associated with risk of MI, levels of VCAM-1 were actually not, with VCAM-1 levels actually similar with and without MI (levels 638 vs 634 ng/mL). I did find a different reference suggesting a potential association (Wallen NH, Eur Heart J 1999, but p value for association was only 0.05).

Have these markers previously been associated with adverse pregnancy outcomes, or is this the first study that has looked at this association? There was only one study cited (ref 28 Chen X et al. PLosOne 2014) that did suggest an association of preterm delivery with elevated sCAM-1 and sVCAM-1 but that was not the adverse outcome being evaluated in this study. Are there associations with stillbirth and SGA?

**Authors Response 1.4:**

There is a study that evaluated Pre-pregnancy Endothelial Dysfunction and birth outcomes (Preterm Birth and SGA Lane-Cordova et al 2018 Pre-pregnancy endothelial dysfunction and birth outcomes: The Coronary Artery Risk Development in Young Adults (CARDIA) Study. Hypertens Res. doi: 10.1038/s41440-018-0017-5. PMID: 29449706; PMCID: PMC6311125) using these markers (e-selectin, ICAM and VCAM). Besides this study, we could not find any other studies that evaluated these specific biomarkers of endothelial dysfunction and associated them with birth outcomes such as stillbirth and SGA.

**Reviewer Comments#2:** This manuscript presents a prospective cohort study that evaluated differences in biomarkers of endothelial function in pregnancy by HIV and ART status as well as the relationship of the endothelial function biomarkers and birth outcomes. Overall, the research question is novel, and the study was well conducted; however, I think the manuscript could be improved in terms of the clarity of the reporting of the methods and the birth outcomes analyzed. My primary concern, which is also acknowledged as a limitation by the authors, is limited statistical power for the adverse birth outcomes examined.

**Authors Response 2:** Thank you for these comments. We agree that statistical power is limited by sample size (particularly among HIV-negative women). This is noted as a limitation in the manuscript.

**Reviewer Comments #2.1** Introduction – The introduction does a nice job laying out the current literature, but the research question is not entirely clear in the last sentence of the section. Based on the methods and results, I think the study had two components to the research question (1) assess the relationship of HIV, ART with the biomarkers and then (2) the relationship of the biomarkers with adverse birth outcomes. It seems part 1 – which takes up a significant proportion of results and discussion is currently missing from the research question.

**Authors Response 2.1:** Thank you for noting this. We have updated the last sentence of the introduction section to read as follows: “We assessed the relationship between (1) HIV, ART and VCAM-1, ICAM-1 and e-selectin, and (2) these biomarkers and adverse birth outcomes.

**Reviewer Comments #2.2** Methods - Lab section –Were there limits of detection per the kits for each biomarker? If yes, please report also how in the analysis how those beyond the limits of detection contributed to the analysis.

**Authors Response 2.2:**

We updated the laboratory methods sections. Measurements outside the linear range were diluted appropriately.

*“We measured plasma concentrations of endothelial dysfunction biomarkers ICAM-1 (detection range 0.3 - 20 ng/mL and sensitivity 0.057) ng/mL, VCAM-1(detection range 6.3 - 200 ng/mL and sensitivity 1.26 ng/mL) and E-selectin (0.1 - 8 ng/mL and sensitivity 0.027 ng/mL) using commercial ELISA kits (R&D Inc, Minneapolis, USA) , following the manufacturer’s instructions”*

**Reviewer Comments #2.3** Methods – it would be important for the methods for stillbirth determination, gestational age dating and birthweight data collection be defined. The exposure methods for biomarkers are clear but how outcome data was collected is not clear.

**Authors Response 2.3:** Thank you for this comment. We have added the following italicized text to the end of the first paragraph of the “Study Population” section: “Gestational age was estimated by the last menstrual period *(which was ascertained using a combination of maternal self-report and written obstetric records that are completed for all pregnant women during antenatal care and labour/delivery, which nearly universally occur during admission to maternity wards). Written obstetric records also served as the source of birthweight and stillbirth data. Small for gestational age was calculated using estimated gestational age and birthweight.”*

**Reviewer Comments #2.4** Preterm birth and Low birthweight –Why were preterm birth, birth weight, low birthweight and other commonly defined pregnancy outcomes not reported? There is certainly existing research and potential links between endothelial dysfunction and preterm birth.

**Authors Response 2.4:** We hypothesized that vascular endothelial dysfunction due to maternal HIV infection (and/or due to ART taken in pregnancy) adversely impacts placental perfusion during pregnancy, leading to increased risk of placenta-mediated pregnancy complications such as fetal growth restriction/small for gestational age and stillbirth**.** We thus focused on stillbirth and small for gestational age as our adverse pregnancy outcomes of interest because these are the outcomes that are most clearly associated with endothelial dysfunction affecting placentation. We have added this text to the end of the Introduction section.

**Reviewer Comments #2.5** Gestation duration, birthweight –Given the sample size, statistical power is a concern as noted by the study team. An analysis of continuous gestational age and birthweight which are commonly analyzed in pregnancy cohorts should be considered. Continuous birthweight-for-gestational age from Intergrowth, which is not as common as birthweight and gestation duration, could also be examined.

**Authors Response 2.5:**

We thank the reviewer for the suggestions.

We have analysed and have also used generalized linear models (GLM) to evaluate the association between continuous birthweight and gestational age and markers of endothelial dysfunction, adjusting for maternal age (continuous) and timing of ART relative to sample collection. We have updated the abstract, statistical analysis section, results sections, accordingly

**Reviewer Comments #2.6** Stillbirth - There were 9 stillbirths in the cohort and as a result this cohort does not seem well positioned to evaluate the relationship between the biomarkers and stillbirth as a ‘primary’ outcome. While acknowledged in the discussion, it seems making no association with stillbirth a key message when there was almost no possibility of finding a difference is challenging and may be misinterpreted. This is in contrast to SGA, for which the sample size is not large, but there was certainly a chance of finding a moderate to large magnitude of effect if there was one.

**Authors Response 2.6:** We agree with these points. We have added the numbers of women experiencing stillbirth and SGA to the abstract (and avoided mentioning lack of association with the pregnancy outcomes in the abstract conclusions, for reasons of low power, particularly for the stillbirth outcome). We have also added the following text to the end of the first paragraph of the Discussion section: “…and we had limited power to evaluate stillbirth as an outcome)”

**Minor** **Comments**

**Minor Comment 1.1** Abstract – it would be helpful to report stillbirth and SGA rates separately to match analysis to be clear it was not a composite endpoint

**Response 1.1:** Stillbirth and SGA rates now reported separately**.**

**Minor Comment 1.2** Abstract and Discussion conclusion – The abstract conclusion focuses on difference by HIV and then the discussion conclusion focuses on birth outcomes. It seems both components are part of the research question and would be important to address in both sections.

**Response 1.2:** Abstract and Discussion sections have been updated to address this comment.

**Minor Comment 1.3** Methods – the order of the analyses presented in the statistical analysis is confusing given the relationship of maternal factors with biomarkers is at the beginning and end of the section with the adverse birth outcomes in the middle.

**Response 1.3:** We modified the order of the Methods section in order to consistently present the analyses with the relationship of biomarkers and maternal factors discussed first, and the relationship of biomarkers and pregnancy outcomes thereafter (in both the Methods and Results sections).

**Minor Comment 1.4** Figure 1 – Please check labels – “ARV before pregnancy sampled before ARV start” – I believe this is women who started ARVs in pregnancy but the sample was collected before they started

**Response 1.4:** That is correct. The labels should be for women who started ART in pregnancy and the sample was collected before vs. after the start of ART. The labels have been updated to read as follows; 1. ART started during pregnancy, sampled before ART initiation, and 2. ART started during pregnancy, sampled after ART initiation**.**

**Minor Comment 1.5** Multiple gestation - were there any twins in the cohort examined? If so, how was this issue addressed in the statistical analysis.

**Response 1.5:** Multiple gestation (i, e,. twins) were excluded from this study analysis. This text has been added in the manuscript methods section under study population.

**Funding**

This was made possible with help from the Harvard University Center for AIDS Research (CFAR), an NIH-funded program (P30 AI060354), which is supported by the following NIH Co-Funding and Participating Institutes and Centers: National Institute of Allergy and Infectious Diseases, National Cancer Institute, Eunice Kennedy Shriver National Institute of Child Health and Human Development, National Heart, Lung, and Blood Institute, National Institute on Drug Abuse, National Institute of Mental Health, National Institute on Aging, National Institute of Diabetes and Digestive and Kidney Diseases, National Institute of General Medical Sciences, National Institute on Minority Health and Health Disparities, National Institute of Dental and Craniofacial Research, Office of AIDS Research, and Fogarty International Center. SM was partially supported through the Sub-Saharan African Network for TB/HIV Research Excellence (SANTHE 2.0), by Bill and Melinda Gates Foundation (INV-033558) and the National Institutes of Health NIH Fogarty International Center K43 TW012350-01.

SL was supported by the National Institutes of Health NIH/ National Institute of Allergy and Infectious Diseases K24 mentoring grant - NIH K24 AI131928. The views expressed in this publication are those of the author(s) and do not necessarily represent the official views of the National Institutes of Health or the Bill and Melinda Gates Foundation.

All funders had no role in the study design, data collection and analysis, decision to publish, or preparation of the manuscript.

There was no additional external funding received for this study

**IRB/Ethics Committees approvals**

The Tshipidi study (from which the de-identified data for this analysis came from) was approved by the Botswana Health Research Development Committee and the Office of Human Research Administration at the Harvard T.H. Chan School of Public Health. Mothers provided written informed consent for study participation.
