# Supplementary material for: Maternal biomarkers of endothelial dysfunction and pregnancy outcomes in women with and without HIV in Botswana
Source: PLoS One. 2023 Feb 23;18(2):e0281910. doi: 10.1371/journal.pone.0281910 (PMC9949641; doi:10.1371/journal.pone.0281910)
Supplement: Attachment — Submitted filename: Response to Reviewers - December 21, 2022.docx [file pone.0281910.s003.docx]

Academic Editor

PLOS one Journal

Dear Ashish KC,

**RE: PONE-D-21-20063R1 “Maternal biomarkers of endothelial dysfunction and pregnancy outcomes in women with and without HIV in Botswana”**

Thank you for the response given to our manuscript titled “*Maternal biomarkers of endothelial dysfunction and pregnancy outcomes in women with and without HIV in Botswana”.*  We have revised the manuscript by addressing point-by-point the comments made by the sole reviewer.

Gaerolwe Masheto

Corresponding Author

Reviewer's Responses to Questions

#6. Review Comments to the Author

Please use the space provided to explain your answers to the questions above. You may also include additional comments for the author, including concerns about dual publication, research ethics, or publication ethics. (Please upload your review as an attachment if it exceeds 20,000 characters)

**Reviewer Comments #6.1:** The reviewer comments have been generally addressed adequately.

The authors now newly note a possible role of e-Selectin and ICAM in lower birthweight and gestation age at delivery at several points in the manuscript. It might be clearly noted that this was regardless of HIV status, given that there were no differences in these parameters by HIV status. So the relevance of these findings are really not HIV-specific but more relate to potential mechanisms of low birth weight and gestational age in general - think this might be made clearer as the paper is focused on differences due to HIV infection.

**Authors Response 6.1:** Thank you for this comment. We agree and we have updated the manuscript to make this clear on the manuscript.

**Reviewer Comments #6.2:** Thank you to the team for your thoughtful response to my comments.

I only have one small comment on the continuous analysis of birth weight and delivery. The Beta is given as -0.07 for example but as a reader I am not clear what the unit is in terms of weight same for the gestation age. As a result the magnitude of the association is not clear.

Is the birthweight kg or grams - is it 70 grams per log increase in log e-selectin? I assume for gestational age its weeks -0.06 weeks per log increase ICAM?

**Authors Response 6.2:** Thank you for the comment. The unit for the birthweight is grams and that for gestational age is weeks. We have edited the manuscript to make this clear for the readers.

**Funding**

This was made possible with help from the Harvard University Center for AIDS Research (CFAR), an NIH-funded program (P30 AI060354), which is supported by the following NIH Co-Funding and Participating Institutes and Centers: National Institute of Allergy and Infectious Diseases, National Cancer Institute, Eunice Kennedy Shriver National Institute of Child Health and Human Development, National Heart, Lung, and Blood Institute, National Institute on Drug Abuse, National Institute of Mental Health, National Institute on Aging, National Institute of Diabetes and Digestive and Kidney Diseases, National Institute of General Medical Sciences, National Institute on Minority Health and Health Disparities, National Institute of Dental and Craniofacial Research, Office of AIDS Research, and Fogarty International Center. Research reported in this publication was supported in part by the Fogarty International Center and the National Institute of Mental Health, of the National Institutes of Health under Award Number D43 TW010543. SM was partially supported through the Sub-Saharan African Network for TB/HIV Research Excellence (SANTHE 2.0), by Bill and Melinda Gates Foundation (INV-033558) and the National Institutes of Health NIH Fogarty International Center K43 TW012350-01.

SL was supported by the National Institutes of Health NIH/ National Institute of Allergy and Infectious Diseases K24 mentoring grant - NIH K24 AI131928. The views expressed in this publication are those of the author(s) and do not necessarily represent the official views of the National Institutes of Health or the Bill and Melinda Gates Foundation.

All funders had no role in the study design, data collection and analysis, decision to publish, or preparation of the manuscript.

There was no additional external funding received for this study

**IRB/Ethics Committees approvals**

The Tshipidi study (from which the de-identified data for this analysis came from) was approved by the Botswana Health Research Development Committee and the Office of Human Research Administration at the Harvard T.H. Chan School of Public Health. Mothers provided written informed consent for study participation.
